# Comprehensive analysis of peptide-coding genes and initial characterization of an LRR-only microprotein in *Marchantia polymorpha*

**DOI:** 10.3389/fpls.2022.1051017

**Published:** 2023-01-19

**Authors:** Haruaki Kobayashi, Kazuaki Murakami, Shigeo S. Sugano, Kentaro Tamura, Yoshito Oka, Tomonao Matsushita, Tomoo Shimada

**Affiliations:** ^1^ Graduate School of Science, Kyoto University, Kyoto, Japan; ^2^ Bioproduction Research Institute, The National Institute of Advanced Industrial Science and Technology, Tsukuba, Ibaraki, Japan; ^3^ Department of Environmental and Life Sciences, University of Shizuoka, Shizuoka-shi, Shizuoka, Japan

**Keywords:** *Marchantia polymorpha*, signaling peptide, small ORF, reproductive induction, LRR protein

## Abstract

In the past two decades, many plant peptides have been found to play crucial roles in various biological events by mediating cell-to-cell communications. However, a large number of small open reading frames (sORFs) or short genes capable of encoding peptides remain uncharacterized. In this study, we examined several candidate genes for peptides conserved between two model plants: *Arabidopsis thaliana* and *Marchantia polymorpha*. We examined their expression pattern in *M. polymorpha* and subcellular localization using a transient assay with *Nicotiana benthamiana*. We found that one candidate, Mp*SGF10B*, was expressed in meristems, gemma cups, and male reproductive organs called antheridiophores. MpSGF10B has an N-terminal signal peptide followed by two leucine-rich repeat (LRR) domains and was secreted to the extracellular region in *N. benthamiana* and *M. polymorpha*. Compared with the wild type, two independent Mp*sgf10b* mutants had a slightly increased number of antheridiophores. It was revealed in gene ontology enrichment analysis that Mp*SGF10B* was significantly co-expressed with genes related to cell cycle and development. These results suggest that MpSGF10B may be involved in the reproductive development of *M. polymorpha*. Our research should shed light on the unknown role of LRR-only proteins in land plants.

## 1 Introduction

Over the last two decades, many biologically active peptides have been identified in land plant species that play critical roles in plant growth and development through molecular genetic studies combined with biochemical analyses. Plant peptides are generally defined as less than 100 amino acids in length. Their structural characteristics can be classified into two major groups (Matsubayashi, 2014; Hirakawa et al., 2017; Stührwohldt and Schaller, 2018). One group is small post-translationally modified peptides characterized by the presence of post-translational modifications and by their small size after proteolytic processing (approximately 5–20 amino acids). The other group comprises cysteine-rich peptides (approximately 40–120 amino acids), characterized by an even number of cysteine residues forming intramolecular disulfide bonds. These plant peptides are often generated by proteolytic processing from larger precursors with N-terminal secretion signal sequences and post-translational modifications. Mature peptides generally function as extracellular ligands in cell-to-cell or long-distance signaling by interacting with their corresponding receptors, leucine-rich repeat receptor-like kinases/proteins (LRR-RLKs/RLPs), on the plasma membrane of target cells.

Since the discovery of systemin in *Solanum lycopersicum* (Pearce et al., 1991), several classes of small secreted peptides have been identified in land plant species. Hormone-like peptides are classified into several families, including CLAVATA3/EMBRYO SURROUNDING REGION-RELATED (CLE), INFLORESCENCE DEFICIENT IN ABSCISSION (IDA), EPIDERMAL PATTERNING FACTOR (EPF), and RAPID ALKALINIZATION FACTOR (RALF) (Gancheva et al., 2019; Furumizu et al., 2021). These peptide families and their receptors are conserved in embryophytes but are rare in green algae (Ghorbani et al., 2015; Bowman et al., 2017; Olsson et al., 2018), indicating that these peptide-signaling pathways evolved in a common ancestor of land plants and served some advantage in terrestrialization (Furumizu et al., 2018; Moody, 2019). In one example, CLE peptides and their receptors are conserved between *Marchantia polymorpha* and *Arabidopsis thaliana* and regulate the size of meristems (Fletcher et al., 1999; Hirakawa et al., 2019; Hirakawa et al., 2020). Many peptide members are encoded as multiple paralogs in most land plant species, which makes it challenging to analyze their characteristics genetically, while *M. polymorpha* has low redundancy in peptide families (Bowman et al., 2017; Furumizu et al., 2021). Furthermore, *M. polymorpha* provides a feasible system for studying small peptides reverse-genetically: for example, gateway binary vectors for *M. polymorpha* (Ishizaki et al., 2015), *Agrobacterium*-mediated transformation (Kubota et al., 2013; Tsuboyama and Kodama, 2018), and CRISPR/Cas9–based genome editing (Sugano et al., 2018).

Although the importance of bioactive peptides in plant cells has been recognized, reverse genetic analysis to search for new peptides has been difficult. This is due to the short sequence length of peptides, which is often judged as noise rather than a gene by conventional gene prediction (Hellens et al., 2016). In plants, an attempt was made to comprehensively search for small open reading frames (sORFs) that are likely to encode peptides. This was made by a powerful gene prediction method in which statistical processing based on the base composition of known protein-coding genes and the transcriptome are combined, and a high rate of false-positive prediction is overcome (Hanada et al., 2010; Hanada et al., 2013). This study identified 7,901 sORFs as candidates for new genes in *A. thaliana*. Among these sORFs, 49 were found to cause morphological changes when overexpressed (Hanada et al., 2013). Several databases for sORFs have recently been established (Hazarika et al., 2017; Chen et al., 2020; Liang et al., 2021). Furthermore, in the Arabidopsis Information Resource (TAIR) database, small coding genes of less than 120 amino acids in length have increased with version updates (Takahashi et al., 2019). These findings suggest that many functional sORFs or peptides in genomes remain uncharacterized involved in various physiological phenomena.

Here, we searched for novel peptide-coding genes conserved between land plant species and analyzed their characteristics using the high-quality dataset of *A. thaliana* and the feasible platform for reverse genetics of *M. polymorpha*.

## 2 Results

### 2.1 Selection of putative peptide-coding genes conserved between *Marchantia polymorpha* and *Arabidopsis thaliana*

**Figure 1:**
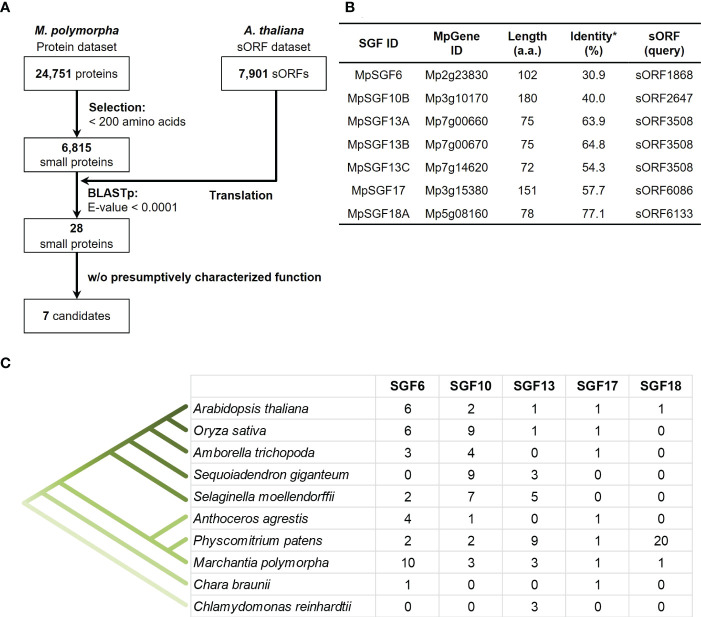
Selection of candidates for *M. polymorpha* peptide-coding genes. **(A)** A pipeline for the selection of peptide candidates. The *A. thaliana* sORF dataset is used as a query for BLASTp analysis. **(B)** A list of selected peptide candidates in *M. polymorpha*. *Percentages of identity between MpSGFs and corresponding sORFs in *A. thaliana* are shown. **(C)** The number of orthologs of SGFs in land plants. Orthologous genes with the same gene family ID (ORTHO05DXXXXXX) to Mp*SGF*s were obtained from Dicots PLAZA 5.0 (Bel et al., 2021; https://bioinformatics.psb.ugent.be/plaza/versions/plaza_v5_dicots/). For *A. thaliana*, sORF genes without AGI codes were included. For *M. polymorpha*, only genes in the latest database, MpTak v6.1, are shown. See **Supplementary Table S3** for more details.

We searched genes in *M. polymorpha* that had high sequence similarity to sORFs in *A. thaliana* to find small genes that are evolutionarily conserved among land plants (datasets of the sORFs are available from https://rdr.kuleuven.be/dataset.xhtml?persistentId=doi:10.48804/SP9WIK). The workflow is shown in **Figure 1A**. From 24,751 proteins in the *M. polymorpha* dataset, we first selected 6,815 small proteins with a length of < 200 amino acids. We then performed a BLASTp search using this small protein dataset as a database and the translated sORFs dataset in *A. thaliana* as a query. After that, we selected 28 genes in *M. polymorpha* encoded putative bioactive peptides (E-value < 0.0001). The search results are listed (**Table 1** and **Supplementary Table S1**). The resulting genes can be classified into 18 groups (collectively designated as “SMALL GENE FAMILIES”; SGF1–SGF18). Among these 28 genes, we focused on 7 Mp*SGF* genes, whose functions cannot be presumed from annotations registered in MarpolBase (https://marchantia.info/; version 5), as candidates without characterized functions (**Figure 1B**, **Table 1**, and **Supplementary Table S1**).

**Table 1:** A list of MpSGFs, containing BLAST results and annotations registered in the database (MarpolBase).

MpSGF ID	MpGene ID	sORF ID (query)	Identity (%)	E-value	Bit score	Functional annotation in MarpolBase
MpSGF1	Mp1g07140^*^	sORF0377	45.614	9.76E-13	54.7	Yes
MpSGF2	Mp6g01390	sORF0615	50.526	2.17E-23	84.3	Yes
MpSGF3	Mp5g15190^*^	sORF1673	64.286	8.87E-06	36.6	Yes
MpSGF4	Mp7g02600	sORF1694	51.948	1.02E-25	87.4	Yes
MpSGF5A	Mp2g20530^*^	sORF1820	87.5	2.17E-20	73.6	–
MpSGF5A	Mp2g20530^*^	sORF4433	87.5	2.17E-20	73.6	–
MpSGF6	Mp2g23830	sORF1868	30.851	5.56E-08	44.3	No
MpSGF7	Mp8g00200	sORF2128	37.313	2.80E-10	49.7	Yes
MpSGF8	Mp4g08500	sORF2161	82.143	8.39E-26	89	Yes
MpSGF9	MpVg01020	sORF2341	36.364	1.57E-05	36.6	Yes
MpSGF10A	Mp1g2534^†^	sORF2647	39.13	5.43E-06	39.3	No
MpSGF10B	Mp3g10170	sORF2647	36.957	7.36E-06	40	No
MpSGF11A	Mp4g19140	sORF2716	38.462	6.37E-11	51.2	Yes
MpSGF11B	Mp4g19080	sORF2716	43.182	1.91E-05	36.2	Yes
MpSGF12	Mp3g15410	sORF3431	67.327	2.43E-44	135	Yes
MpSGF13A	Mp7g00660	sORF3508	63.889	5.71E-33	105	No
MpSGF13B	Mp7g00670	sORF3508	64.789	1.94E-30	98.6	No
MpSGF13C	Mp7g14620	sORF3508	54.286	1.44E-23	81.3	No
MpSGF14	Mp6g09670	sORF3693	43.548	1.36E-11	55.8	Yes
MpSGF15	Mp1g23010^*^	sORF4434	47.619	1.07E-09	47	–
MpSGF16A	Mp4g14630	sORF4935	33.028	4.53E-11	53.5	Yes
MpSGF16B	Mp7g13800	sORF4935	33.945	7.62E-10	50.1	Yes
MpSGF16C	Mp8g16530	sORF4935	29.358	4.51E-07	42.4	Yes
MpSGF16D	Mp5g00590	sORF4935	28.037	8.55E-07	41.6	Yes
MpSGF16E	Mp3g23830	sORF4935	34.783	8.49E-06	38.9	Yes
MpSGF16F	Mp3g07430	sORF4935	29.268	9.90E-05	35.8	Yes
MpSGF17	Mp3g15380	sORF6086	57.692	2.59E-14	61.6	No
MpSGF18A	Mp5g08160	sORF6133	77.143	2.30E-13	53.9	No
MpSGF18B	Mp4g23800^*^	sORF6133	74.286	1.39E-12	52	–

* Deleted in newer database ver. 6 (MpTak v6.1; released on 2021.3.15). ^†^non-canonical protein-coding gene (e.g., non-AUG start codon). See **Table S1** for detailed information.

The selected seven SGF genes are well conserved among land plants, and some are found in green algae (**Figure 1C**). Multiple sequence alignments and molecular phylogenetic analysis were performed (**Supplementary Figures S1**, **S2**). The amino acid sequences of SGF6 and SGF10 are conserved across the entire length of the proteins in various plant species. SGF13 homologs are enriched in non-flowering plants and the green alga *Chlamydomonas reinhardtii*. They are conserved over the entire sequence. Some homologs have an extended N-terminus. SGF17 homologs are found from the Charophyceae *Chara braunii* to the flowering plants, and the C-terminal region is highly conserved. SGF18 homologs are only found in *A. thaliana*, *M. polymorpha*, and *Physcomitrium patens* and are highly enriched in *P. patens*. The middle region is well conserved among SGF18 orthologs.

### 2.2 Expression analysis and subcellular localization of MpSGFs

**Figure 2:**
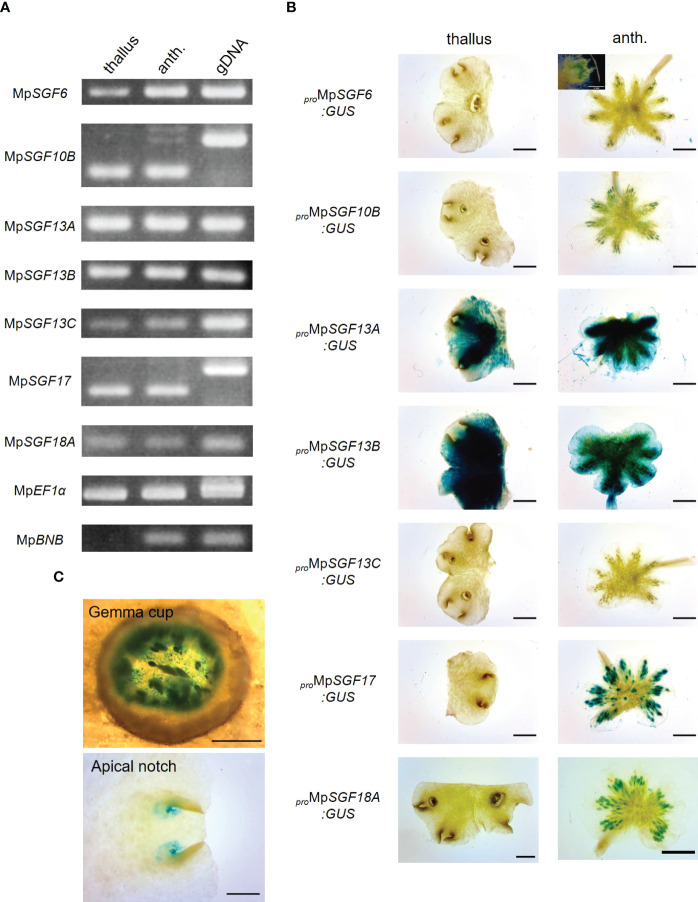
Expression patterns of Mp*SGF*s in *M. polymorpha*. **(A)** RT-PCR in 2-week-old thallus (thallus) and antheridiophore (anth.). Mp*EF1α* is used as an internal control. Mp*BNB* is used as a marker gene for antheridiophore (Yamaoka et al., 2018). **(B)** Representative images of the GUS reporter assay for expression patterns of MpSGFs. Two-week-old thalli and antheridiophores were observed for the visualization of promoter activities. Bars = 2 mm. For *_pro_* Mp*SGF6:GUS*, a magnified image around antheridia is shown in the inset, bar = 1 mm. **(C)** Magnified images of GUS signal in the bottom of gemma cups and apical notches of *_pro_* Mp*SGF10B:GUS* plants. Bars = 0.5 mm.

Next, we examined expression pattern of the selected 7 Mp*SGF* genes. It was revealed in reverse transcription-PCR (RT-PCR) analyses that the expression of all Mp*SGF* genes was detected in thalli and antheridiophores, corresponding to vegetative organs and reproductive organs, respectively. These results indicate that these MpSGFs function in both vegetative and reproductive states in *M. polymorpha* (**Figure 2A**). The results of RT-PCR were consistent with public transcriptome data (Kawamura et al., 2022). However, the expression levels of each gene varied between organs (**Supplementary Figure S3**).

To investigate the spatial expression pattern of the selected Mp*SGF*s in planta, we observed their promoter activities using transgenic plants harboring the ß-glucuronidase (GUS) reporter. We introduced the GUS reporter constructs with 4–5 kb upstream regions of coding sequence (CDS) into male and female wild-type plants Takaragaike-1 (Tak-1) and Takaragaike-2 (Tak-2), respectively. Two- or 3-week-old thalli and gametophores were sampled and stained as vegetative and reproductive organs, respectively (**Figure 2B** and **Supplementary Figure S4**).

The GUS activity of *_pro_* Mp*SGF6:GUS* in male plants was observed in antheridiophores, especially around antheridia, but not in thalli. In female plants, no signal was observed in thalli and archegoniophores. The GUS activity was specifically observed around apical notches, bottoms of gemma cups, and in gametophores in both male and female *_pro_* Mp*SGF10B:GUS* lines (**Figure 2C**). Intense GUS activities were detected in thalli and antheridiophores in male Mp*SGF13A* and Mp*SGF13B* plants, much weaker activity was found in whole tissues of female plants, except for the gemma cups. No GUS activity was observed in male and female *_pro_* Mp*SGF13C:GUS* lines. The GUS signal was specifically observed in antheridia and archegonia in *_Pro_* Mp*SGF17:GUS* lines. The GUS activity of *_pro_* Mp*SGF18A:GUS* lines was observed in antheridiophores, especially around antheridia, but not in thalli and archegoniophores. These observations are consistent with public transcriptome data, in which the expression level of Mp*SGF18A* is increased in the “sperm cell” (**Supplementary Figure S3**).

**Figure 3:**
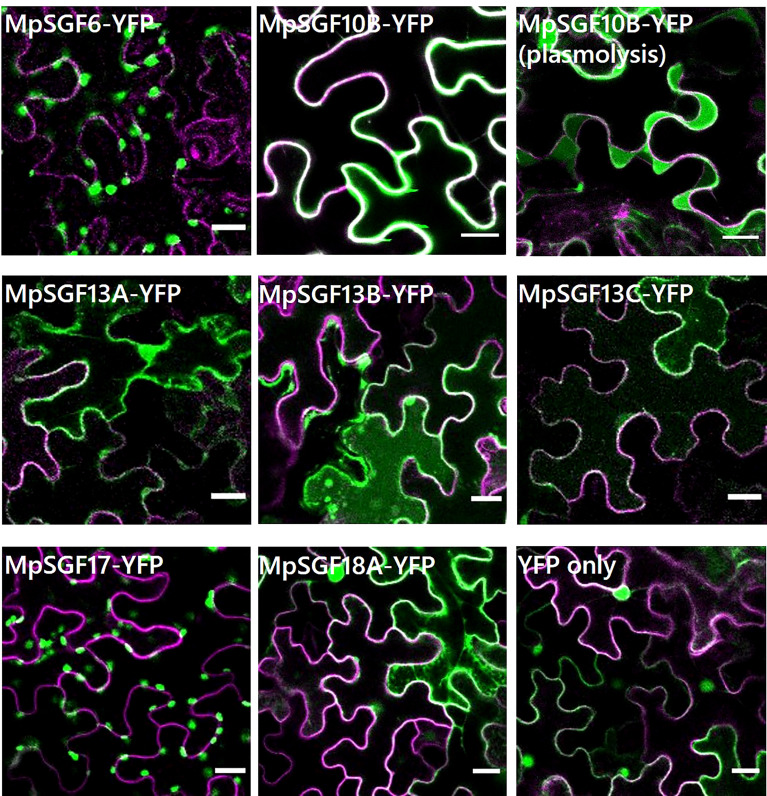
Subcellular localization of MpSGFs in *N. benthamiana*. Confocal fluorescence microscopy images of MpSGF-YFP transiently expressed in *N. benthamiana*; MpSGF6, MpSGF10B under control (water) and hypertonic conditions, MpSGF13A, MpSGF13B, MpSGF13C, MpSGF17, MpSGF18A, and YFP only. The plasma membrane, which was visualized by mRFP-LTI6b, and YFP were shown in magenta and green, respectively. Bars = 20 µm.

We performed a transient expression assay for a MpSGF and yellow fluorescent protein fusion proteins (MpSGF-YFP) using *Nicotiana benthamiana* to determine the subcellular distribution of MpSGFs (**Figure 3**). MpSGF6-YFP was co-localized with chlorophyll (**Supplementary Figures S5A, C**). While MpSGF10B-YFP was detected in the periphery of epidermal cells and appeared to overlap with the plasma membrane, the signal was visualized in an extracellular region after plasmolysis treatment, suggesting that MpSGF10B can be transported to the extracellular region. MpSGF13A-YFP, MpSGF13B-YFP, and MpSGF13C-YFP were ambiguously observed in the cytosolic compartment. MpSGF17-YFP was co-localized with chlorophyll (**Supplementary Figures S5B ,D**). MpSGF18A-YFP was observed in a cytosolic compartment.

### 2.3 Functional analysis of Mp*SGF10B* in *Marchantia polymorpha*

**Figure 4:**
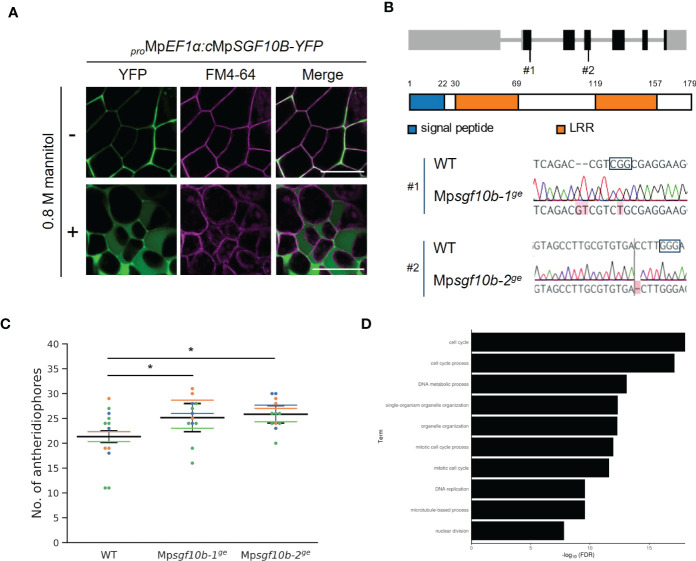
Reverse genetic analysis of MpSGF10B. **(A)** Subcellular localization of MpSGF10B-YFP constitutively expressed in *M. polymorpha*. Three-day-old gemmalings were observed. The plasma membrane was visualized with FM4-64 (magenta). Shown data are representative ones from 5 independent transgenic lines. Bars = 50 µm. **(B)** Gene structures and genotypes of Mp*sgf10b* mutants using CRISPR/Cas9. (top) Structure of MpSGF10B/Mp3g10170 locus with the positions of the designed guide RNAs (#1 and #2). Black boxes, gray lines, and gray boxes show coding exons, introns, and untranslated regions. (Middle) The predicted product of MpSGF10B is shown. (Lower) Sequences of genome editing alleles are shown. The PAM sequence is shown in enclosed characters. Edited bases are indicated with red shades. **(C)** The number of antheridiophores in an individual plant. *n* = 3, 3, and 5 for all lines from three independent experiments, respectively (indicated in distinct colors). Horizontal lines indicate the average numbers grouped by the experiments. Black lines show the total means ± SD. *P < 0.05, Dunnett’s test against wild type (WT). **(D)** GO enrichment analysis of highly co-expressed genes with Mp*SGF10B*. The 10 high-ranking biological processes are shown at a 1% false discovery rate (FDR).

We confirmed that MpSGF10B-YFP was also secreted to the extracellular region in *M. polymorpha* (**Figure 4A**). Thus, we focused on MpSGF10B as a candidate for a secreted bioactive peptide, and further analyses were performed. Mp*SGF10B* encodes a protein with an N-terminal signal peptide, followed by two LRR motifs (**Figure 4B**). We generated Mp*sgf10b^ge^* mutants using the CRISPR/Cas9 system to examine the function of the MpSGF10B in *M. polymorpha*. Mp*sgf10b-1^ge^* and Mp*sgf10b-2^ge^* harbor frameshift mutations that generate early stop codons after the predicted signal peptide sequence and in the middle of the sequence, respectively (**Figure 4B**). In the vegetative growth phase, 2-week-old Mp*sgf10b^ge^* mutants showed no significant differences in thallus size and the number of apical notches compared to the wild type (**Supplementary Figure S6**). However, in the subsequent reproductive phase, we found that the number of antheridiophore was slightly increased in both Mp*sgf10b^ge^* mutants after 3-week induction (**Figure 4C**). It is suggested in these results that Mp*SGF10B* might function in the reproductive phase in the liverwort.

We investigated the effect of Mp*SGF10B* mutations on reproductive growth by putting gemmae under reproductive conditions. After 10-day induction, no apparent abnormalities were observed in the branching frequency of thalli and emerging time of gametangiophores in Mp*sgf10b* mutants (**Supplementary Figure S7**). Under the same reproductive condition, the overexpression lines of Mp*SGF10B-YFP* developed slightly fewer antheridiophores than the wild type. Still, there was no statistically significant difference (**Supplementary Figure S8**).

Next, we examined the functional categories of co-expressed genes with Mp*SGF10B* by gene ontology (GO) to infer the functional categories of Mp*SGF10B* at the molecular level. We calculated the R-values (Pearson’s coefficient of correlation) of expression intensities between Mp*SGF10B* and all annotated genes in various organs and conditions. We then defined those annotated genes with the 1% high-ranking R-values as co-expressed genes (Hanada et al., 2013). The over-represented functions of the co-expressed genes were related to cell cycle and development at lower FDR values among the GO categories of biological processes (**Figure 4D** and **Supplementary Table S2**). This was consistent with the above-mentioned reporter assay, since the promoter activity of Mp*SGF10B* was detected around the apice and the bottom of gemma cups, in which cell division was taking place for the growth and production of gemmae, respectively (**Figure 2C**). Collectively, these data suggest that MpSGF10B may be related to cell cycle or cell division at the molecular level.

Since the promoter activity of Mp*SGF10B* was observed in gemma cups and around apical notches (**Figure 2C**), in which auxin-regulated genes are highly expressed (Ishizaki et al., 2012), we presumed that Mp*SGF10B* could be involved in auxin signaling. However, the expression levels of two auxin-responsive genes, Mp*WIP* and Mp*EXP* (Kato et al., 2017), were not altered in Mp*sgf10b^ge^* mutants compared to the wild type (**Supplementary Figure S9**). Furthermore, the expression level of Mp*SGF10B* was also unchanged by auxin dosage according to public transcriptome analyses (**Supplementary Figure S3**). Thus, its contribution to auxin signaling might be little or null.

## 3 Discussion

Over the past few decades, various peptide-coding genes have been predicted in *A. thaliana* (Hanada et al., 2007; Hanada et al., 2013). Since many bioactive peptides tend to be conserved among land plant species, it is reasonable that bioactive peptides can be detected by comparing evolutionally distant plant species (e.g., bryophytes and flowering plants). We investigated novel peptide-coding genes conserved between *A. thaliana* and *M. polymorpha* through a BLAST-based similarity search followed by expression analyses in this study.

### 3.1 Identification and characterization of SGF genes in *Marchantia polymorpha*

It was revealed in our phylogenetic analyses that the selected SGFs, except for SGF18, were conserved in various land plant species (**Figure 1C**; **Supplementary Figures S1**, **S2**). It was suggested in previous analyses that peptide-coding genes have low redundancy in bryophytes and high copy numbers in vascular plants (Bowman et al., 2017; Olsson et al., 2018; Furumizu et al., 2021). However, we found that orthologous genes of SGF13 and SGF17 were mainly enriched in bryophytes. One possibility is that SGF13 and SGF17 gene families might be more adaptive in the life cycle of bryophytes. Another possibility is that many peptide-coding genes remained unannotated even in the latest database of *A. thaliana* (Takahashi et al., 2019; Fan et al., 2022). Recently, comprehensive studies have been performed to identify novel functional peptides in land plants (Fesenko et al., 2019; Wang et al., 2020; Liang et al., 2021). Annotations of small peptide-coding genes will be expanded by these analyses in the future. Thus, SGFs classified in this study can be more conserved in land plant species than we reported here.

Bioactive peptides in plants are generally secreted out of cells and function as extracellular ligands in cell-to-cell signaling by interacting with their corresponding receptors on the plasma membrane of target cells. MpSGF10B possessed an N-terminal signal peptide and was secreted into the extracellular region (**Figures 3B, C**, **4A**). However, an N-terminal signal peptide is not necessary to act as an apoplastic signaling ligand. For example, AtPROPEP1 is localized in the cytosolic compartment before maturation, and its mature form, AtPep1, is generated upon tissue damage and released into the apoplast region (Hander et al., 2019). Therefore, the possibility remains that the other MpSGFs, such as cytosolic MpSGF13A, MpSGF13B, MpSGF13C, and MpSGF18A, function as signaling ligands extracellularly in some cases.

### 3.2 MpSGF10B encodes an extracellular LRR-only protein

We showed that the LRR-containing protein MpSGF10B was secreted to the extracellular region in this study (**Figures 3B, C**). LRR proteins in plants act in various biological processes. According to the subcellular localization and structure, LRR proteins are classified into three groups: LRR-RLKs/RLPs at the plasma membrane, intracellular nucleotide-binding site (NBS)-LRRs, and extracellular LRR-only proteins (Wang et al., 2011). MpSGF10B may belong to the extracellular LRR-only protein group.

LRR-RLKs/RLPs and NBS-LRRs have been well studied and act in plant immune responses, such as various receptors and R-genes (Dievart et al., 2020; Sett et al., 2022). In contrast, extracellular LRR-only proteins have been poorly reported. A well-known LRR-only protein, polygalacturonase inhibitor 1 (PGIP1), participates in disease resistance by inhibiting the activity of fungal pectin-degrading enzymes (Cheng et al., 2021). *Arabidopsis* PGIPs have an N-terminal signal peptide and LRR motifs (PGIP1: Q9M5J9, PGIP2: Q9M5J8 in the UniProt database). Therefore, PGIPs and MpSGF10B have similar features. However, there are some differences between MpSGF10B and rice PGIPs, which are anchored to the plasma membrane or cell wall (Wang et al., 2015) and possess as many as 10 repeats of the LRR motif. It is reported that canonical LRR-only proteins contained 7–13 repeats (Wang et al., 2011). Since MpSGF10B has fewer LRR motifs and is smaller than canonical LRR proteins, it can be considered a non-canonical LRR-only protein (**Figure 4B**). As such non-canonical LRR-only proteins, NTCD4 (F4JNB0 in the UniProt) and LRRop-1 (Q9FPJ5), have been reported in *A. thaliana*. NTCD4 consists of 153 amino acids and interacts with MoNLP1, a microbe-associated molecular pattern, to induce cell death. NTCD4 is secreted in the extracellular region (Chen et al., 2021). LRRop-1, an LRR-only protein of 218 amino acids, has been suggested to contribute to ABA signaling and is localized in the ER (Ravindran et al., 2020).

Several repeats of LRR motifs, which are generally involved in protein-protein interaction, form a twisted super-helical or a small arc-shaped structure (Kobe and Kajava, 2001; Chakraborty et al., 2019). Therefore, it is unlikely that a small number of LRR units can function as canonical receptors. Further studies are necessary to verify the molecular function of MpSGF10B, whether it acts as a coreceptor that assists other receptors or a ligand for signal transduction or serves an entirely different function.

### 3.3 MpSGF10B might be involved in the development of reproductive organs in *Marchantia polymorpha*

We found promoter activity of Mp*SGF10B* in the following organs in *M. polymorpha*: bottom of gemma cups, surrounding areas of the meristem, and antheridia (**Figures 2B, C**). These expression patterns seem to overlap largely with the accumulation patterns of auxin (Ishizaki et al., 2012). Auxin is proposed to control the development of various organs in *M. polymorpha*, including gemmae formation and the branching frequency of thalli (Kato et al., 2020), through regulating the expression pattern of auxin-responsive genes. However, the expression levels of two auxin-responsive genes were not altered in the Mp*sgf10b* mutants (**Supplementary Figure S9**). Consistently, the Mp*sgf10b* mutants did not show any phenotypic changes, which were expected when perturbing the auxin signal (**Supplementary Figure S7**). Furthermore, the expression of Mp*SGF10B* is likely independent of the auxin signal (**Supplementary Figure S3**). Thus, Mp*SGF10B* may function independently of or weakly associated with auxin signaling.

Here, we reported that Mp*SGF10B* might be involved in the induction of reproductive organs in *M. polymorpha* (**Figure 4C** and **Supplementary Figures S8**). In reproductive induction in liverworts, far-red light and circadian rhythms of long-day are key conditions, and these environmental stimuli are sensed and transduced by various signaling genes including Mp*BNB*, which acts as a master regulator of reproductive development (Kohchi et al., 2021; Yamaoka et al., 2021). Homologous modules are involved in light response and gametogenesis in *A. thaliana*, suggesting the conserved roles of these signaling modules among land plant species (Kohchi et al., 2021; Yamaoka et al., 2021). In this study, we found that relevant orthologs of Mp*SGF10B* are conserved in land plant species but not in algae (**Figure 1C** and **Supplementary Figures S1**, **S2**). Unfortunately, this time we could not come close to a clear function of MpSGF10. Further analyses are needed to determine the physiological function of Mp*SGF10B* and its orthologs in *M. polymorpha* and other plant species.

## 4 Materials and methods

### 4.1 Plant materials and growth conditions

*M. polymorpha* male and female accessions, Tak-1 and Tak-2, respectively, were used as the wild type in this study. Plants were axenically grown on half-strength Gamborg’s B5 medium pH 5.7 containing 1% agar and 1% sucrose at 22°C under 50–60 μmol photons m^−2^ s^−1^ continuous white light. For reproductive induction, plants were incubated under long-day conditions (light: 16 h, dark: 8 h) supplemented with continuous far-red light at 18°C emitted from diodes (IR LED STICK 18W, Namoto).

### 4.2 Multiple sequence alignments and phylogenetic analysis

The sequences of orthologous genes, which were assigned the same ortholog IDs as the Mp*SGF*s, were retrieved from dicots PLAZA 5.0 (**Supplementary Table S3**). Briefly, SGF6, SGF10, SGF13, SGF17, and SGF18 correspond to ORTHO05D000774,ORTHO05D001118, ORTHO05D007973, ORTHO05D009227, and ORTHO05D009796, respectively. Multiple sequence alignments were performed using stand-alone MAFFT (v7.490) with the L-INS-i method. The alignments were visualized using the LATEX package, TEXshade. The alignment gaps were removed using TrimAl (v1.4) with the “-automated1” method. Substitution models were evaluated with ModelTest-NG (v0.1.6). Phylogenetic trees were generated using RAxML-NG (v0.9.0), with the substitution models of JTT+G4m, LG+I+G4m, LG+G4m, LG+I+G4m, and JTT+I were used for SGF6, SGF10, SGF13, SGF17, and SGF18, respectively. We performed bootstrap analyses with 1,000 replicates for each analysis to assess the statistical support for the topology.

### 4.3 RNA extraction and RT-PCR

Thalli or antheridiophores frozen in liquid nitrogen were ground with a mortar and a pestle into fine powder. Total RNA was extracted from the tissue powder using the RNeasy Plant Mini Kit (Qiagen). cDNA was synthesized using ReverTra Ace reverse transcriptase (Toyobo). Amplification was performed with GoTaq DNA polymerase (Promega).

### 4.4 Plasmid construction

Primers used in this study are listed in **Supplementary Table S4**. For the promoter GUS constructs, approximately 5,000 bp upstream sequences flanked the start codons of the MpSGFs were amplified from Tak-1 genomic DNA using PrimeSTAR GXL polymerase (Takara Bio) and cloned into a linearized pENTR1A vector using In-fusion (Takara Bio). Inserts were transferred into pMpGWB104 vectors (Ishizaki et al., 2015) using a Gateway LR Clonase II enzyme mix (Thermo Fisher Scientific). For the construction of the transient expression assay, total RNA was extracted from Tak-1 thalli, and cDNA was synthesized. The CDSs of the MpSGFs were amplified from the cDNA using PrimeSTAR GXL polymerase. A DNA fragment of modified Venus (YFP-4×Gly), the fluorescent protein with four Gly-residues at its C-terminus, was amplified from the gateway vector used previously (Sugano et al., 2010). Three fragments of a MpSGF CDS, a YFP-4×Gly CDS, and a linearized pENTR1A backbone were fused using In-fusion. The entry vector was transferred to a binary vector, pGWB602 (Nakamura et al., 2010), with a Gateway LR Clonase II enzyme mix. A genomic fragment of *Arabidopsis* LTI6b was amplified and fused with pENTR1A using In-fusion and transferred to pGWB655 with a Gateway LR Clonase II enzyme mix to construct mRFP-LTI6b. For the constitutive expression of MpSGF10B-YFP in *Marchantia*, the MpSGF10B-YFP in pENTR1A mentioned above was used as an entry vector. The entry vector was transferred to a binary vector pMpGWB103 (Ishizaki et al., 2015) with a Gateway LR Clonase II enzyme mix. For genome editing, guide RNAs were designed at two positions for each target gene using the CRISPR-Cas9 system (Sugano et al., 2018). Double-stranded DNAs corresponding to the guide RNA sequences were generated and inserted into AarI-digested pMpGE013 by ligation reaction using Mighty Mix (Takara Bio).

### 4.5 Generation of transgenic lines

*M. polymorpha* transformants were obtained by *Agrobacterium*-mediated transformation from regenerating thalli using the AgarTrap method (Tsuboyama and Kodama, 2018). Two wild-type accessions, Tak-1 (male) and Tak-2 (female), were used as genetic backgrounds. Transgenic plants were selected with the above-mentioned medium and were supplemented with 10 µg/mL hygromycin. The transformants were clonally purified through gemmae generations. To examine the target loci of genome editing lines, genomic DNAs were extracted from G1-generation plants produced from the asexual reproduction of T1 plants, and then the PCR products were amplified using GoTaq DNA polymerase, followed by direct sequencing (Sugano and Nishihama, 2018).

### 4.6 GUS staining

Transgenic plants were fixed with 90% acetone at 4°C and then stained in GUS staining solution (100 mM sodium phosphate buffer pH 7.4, 0.5 mM potassium ferrocyanide, 0.5 mM potassium ferricyanide, 10 mM EDTA, 0.1% Triton X-100, and 0.5 mg/ml 5-bromo-4-chloro-3-indolyl-b-D-glucuronic acid) overnight at 37°C in the dark for GUS staining. The GUS-stained samples were cleared with 70% ethanol and chloral hydrate solution.

### 4.7 Transient expression assay in *Nicotiana benthamiana*

The *Agrobacterium tumefaciens* GV3101 strain was transformed with plasmids harboring proteins of interest (POIs) and grown in LB media containing spectinomycin (50 µg/mL). Cultures grown for about 24 h at 28°C with rotation at 220 rpm were used to transform *N. benthamiana* leaves. POI-containing bacteria were centrifuged for 15 min at 2,800 × *g* and resuspended in water to 4-fold dilution for infiltration. The cells were infiltrated into the abaxial side of *N. benthamiana* leaves. The infiltrated plants were incubated under dark conditions for 2 days. For imaging, the leaves were infiltrated with water (control) or 0.8 M D-mannitol solution (plasmolysis) using a 1 mL syringe, and leaf disks were harvested immediately.

### 4.8 Confocal fluorescent microscopy

The fluorescent images were inspected under a confocal laser scanning microscope (LSM780; Zeiss) using a 514- or 561-nm laser line. We used filter sets of a 519/549 nm band-pass filter for YFP fluorescence, a 583/633 nm for mRFP, and a 571/645 nm for FM4-64. Fresh samples were mounted in water (control) or 0.8 M D-mannitol solution (plasmolysis) and imaged immediately. For gemmalings, the plasma membrane was stained with 10 µg/ml FM4-64 (Invitrogen). These channel images were processed using ImageJ (Fiji; Schindelin et al., 2012).

### 4.9 Phenotypic observation for Mp*sgf10b^ge^* and Mp*SGF10B-YFP* overexpressor

Growth phenotypes were monitored in plants after reproductive induction for 10–30 days under long-day conditions (light: 16 h, dark: 8 h) supplemented with continuous far-red light at 18°C with or without preculture for two weeks at 22°C under continuous white light. According to the developmental stages of antheridiophores (Higo et al., 2016), antheridiophores of above stage 1, namely “button-like” receptacles, were counted. The number of branches was calculated based on the trajectory of antheridiophores and apical notches in time-lapse images. Apical notches were defined as notches without gametangiophores. The area of thalli was measured with the “color threshold” tool of ImageJ.

### 4.10 Co-expression and GO enrichment analyses

Publicly available fastq data from RNA-Seq libraries were obtained from the sequence read archive in NCBI. All libraries used for analyses were performed in triplicate (except for antheridia duplicates). Fastq sequence data were mapped onto the reference genome of *M. polymorpha* (version 6.1) using STAR (Dobin et al., 2013), and the number of fragments was counted and normalized by transcripts per million (TPM) with RSEM (Li and Dewey, 2011). Accession numbers include: 11-day thalli (DRR050343, DRR050344, and DRR050345), Archegoniophore (DRR050351, DRR050352, and DRR050353), Antheridiophore (DRR050346, DRR050347, and DRR050348), Antheridia (DRR050349, and DRR050350), apical cell (SRR1553294, SRR1553295, and SRR1553296), 13d-Sporophyte (SRR1553297, SRR1553298, and SRR1553299), Sporelings 0 h (SRR4450262, SRR4450261, and SRR4450260), 24hr-Sporeling (SRR4450266, SRR4450265, and SRR4450259), 48hr-Sporeling (SRR4450268, SRR4450264, and SRR4450263), 72hr-Sporeling (SRR4450267, SRR4450258, and SRR4450257), 96hr-Sporeling (SRR4450256, SRR4450255, and SRR4450254), thalli-mock (SRR5905100, SRR5905099, and SRR5905098), and 2,4-D 1h (SRR5905097, SRR5905092, and SRR5905091), plants infected with *P. palmivora* of 2 dpi (SRR7977547, SRR7977549, and SRR7977550), and mock-inoculated plants of 2 dpi (SRR8068335, SRR8068336, and SRR8068340) (Carella et al., 2018; Flores-Sandoval et al., 2018). Pearson’s correlation coefficients (PCCs) were calculated for the TPM of MpSGF10B and all genes using the R application. The top 1% (182 genes) with high PCC values were selected as co-expressed genes with MpSGF10B. These gene IDs were converted to those in assembly version 3 using the “convert ID” tool in MarpolBase (https://marchantia.info/). Then, GO enrichment analysis was performed using the Plant Transcriptional Regulatory Map (PlantRegMap, http://plantregmap.gao-lab.org/).

### 4.11 Statistical analysis

Statistical analyses were performed in R version 4.0.3. One-way ANOVA and one-tailed Dunnett’s test in package “multicomp” were used for multiple comparisons of means grouped by technical replicates.

## Data availability statement

The datasets presented in this study can be found in online repositories. The names of the repository/repositories and accession number(s) can be found in the article/**Supplementary Material**.

## Author contributions

HK and TS conceived and designed the research in general; HK performed most of the experiments and analyzed the data; KM generated a construct for visualizing plasma membranes; SSS supported data analysis; KT, YO, and TM supervised the experiments; HK and TS wrote the manuscript. All authors contributed to the article and approved the submitted version.
