# Supplementary material for: Comprehensive analysis of peptide-coding genes and initial characterization of an LRR-only microprotein in Marchantia polymorpha
Source: Front Plant Sci. 2023 Jan 19;13:1051017. doi: 10.3389/fpls.2022.1051017 (PMC9901580; doi:10.3389/fpls.2022.1051017)
Supplement: Supplementary file 1 [file Presentation_1.pdf]

Supplementary Material

1 Supplementary Figures and Tables

1.1 Supplementary Figures

SGF6: ORTHO05D000774

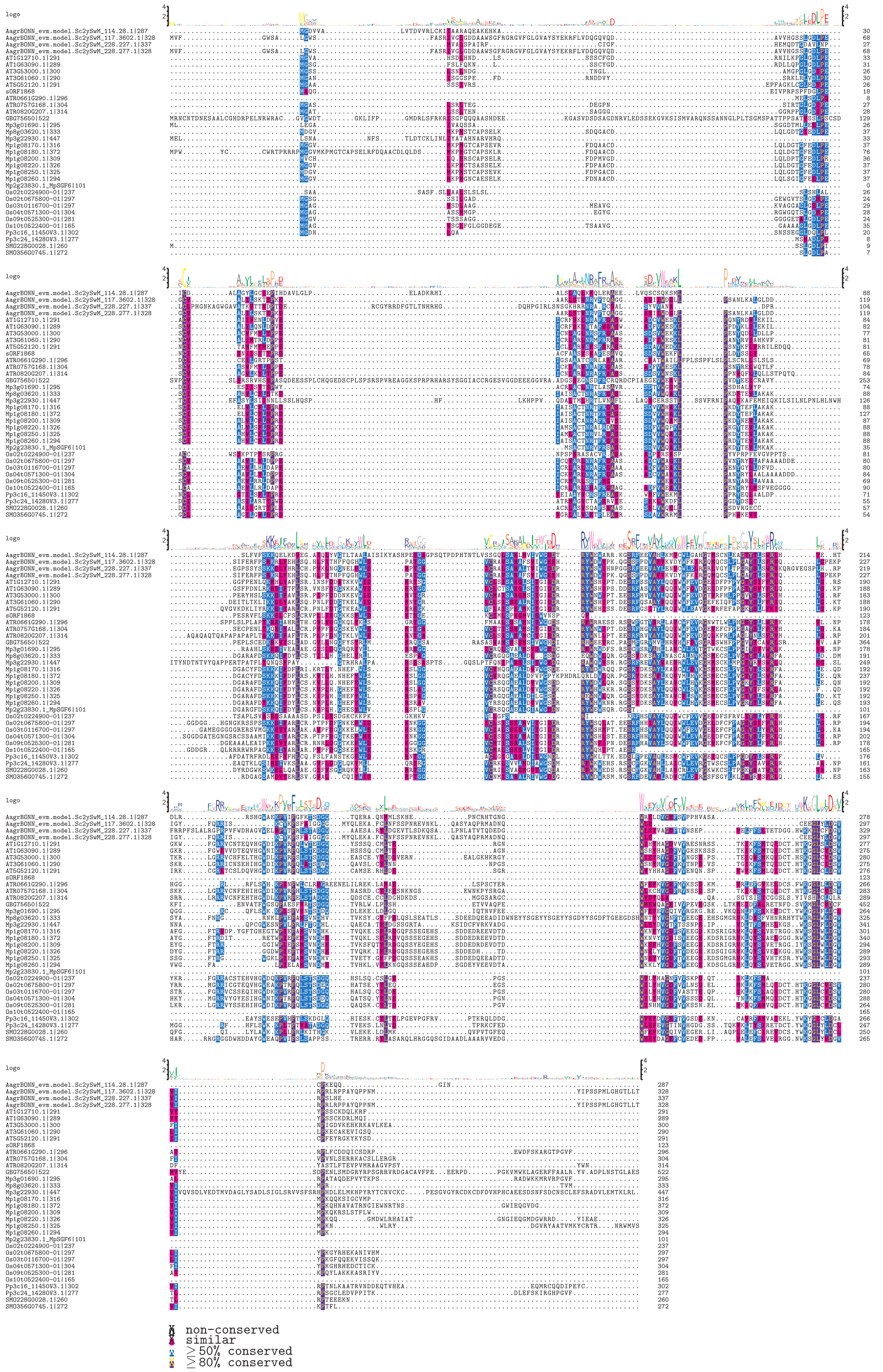

(Continued on next page)

SGF10: ORTHO05D001118

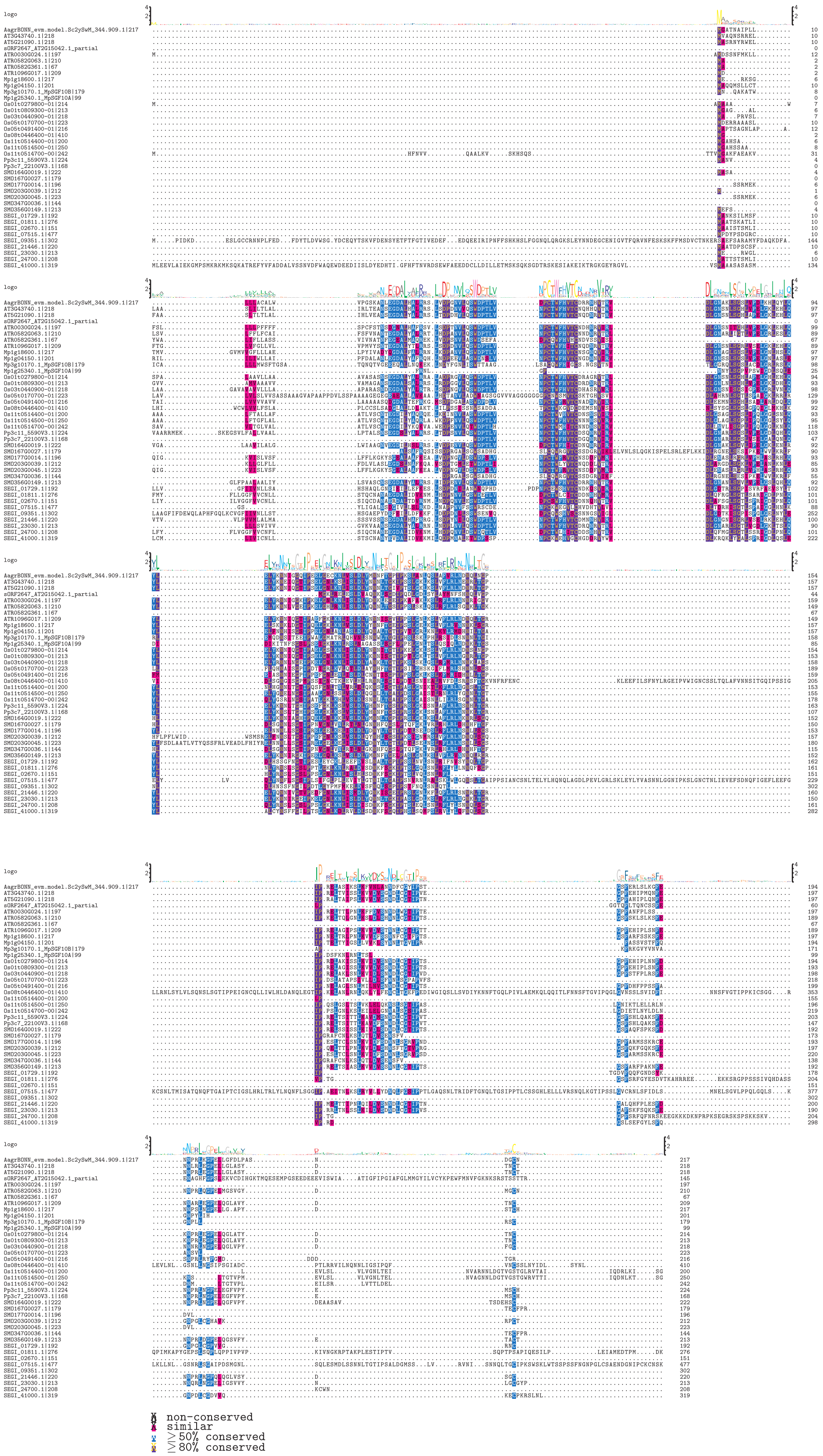

(Continued on next page)

SGF13: ORTHO05D007973

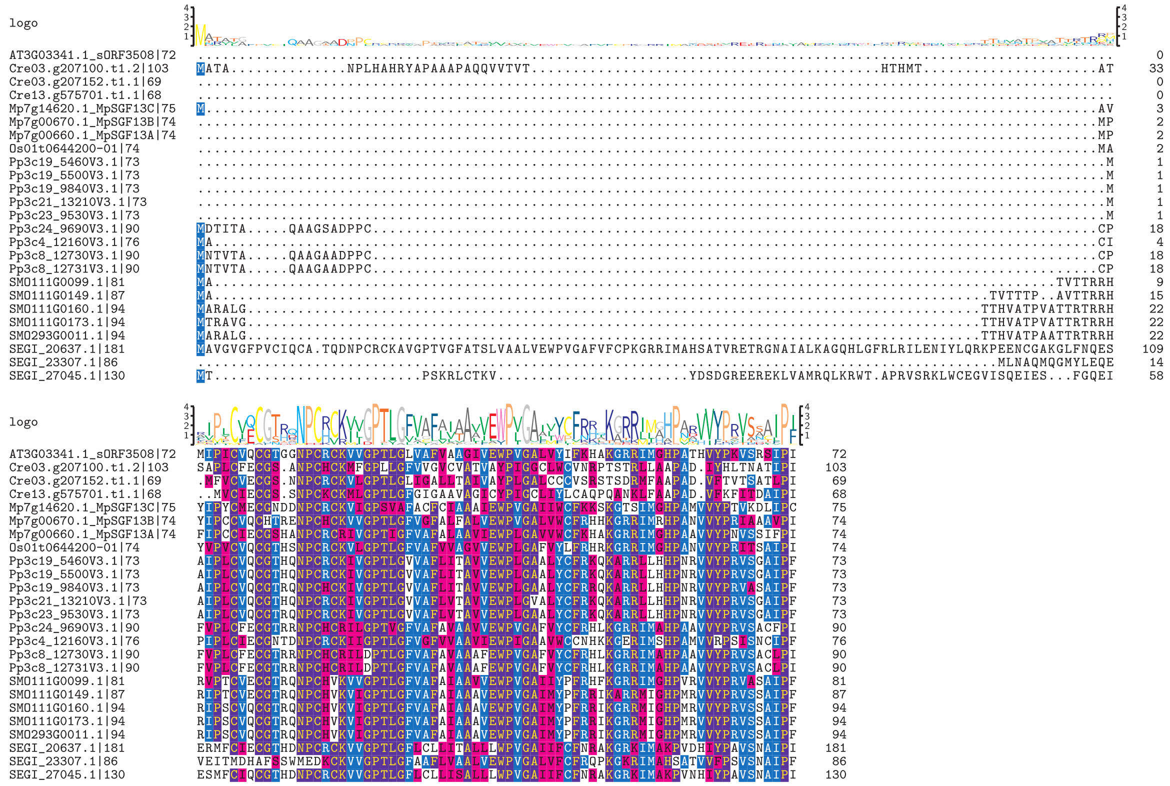

SGF17: ORTHO05D009227

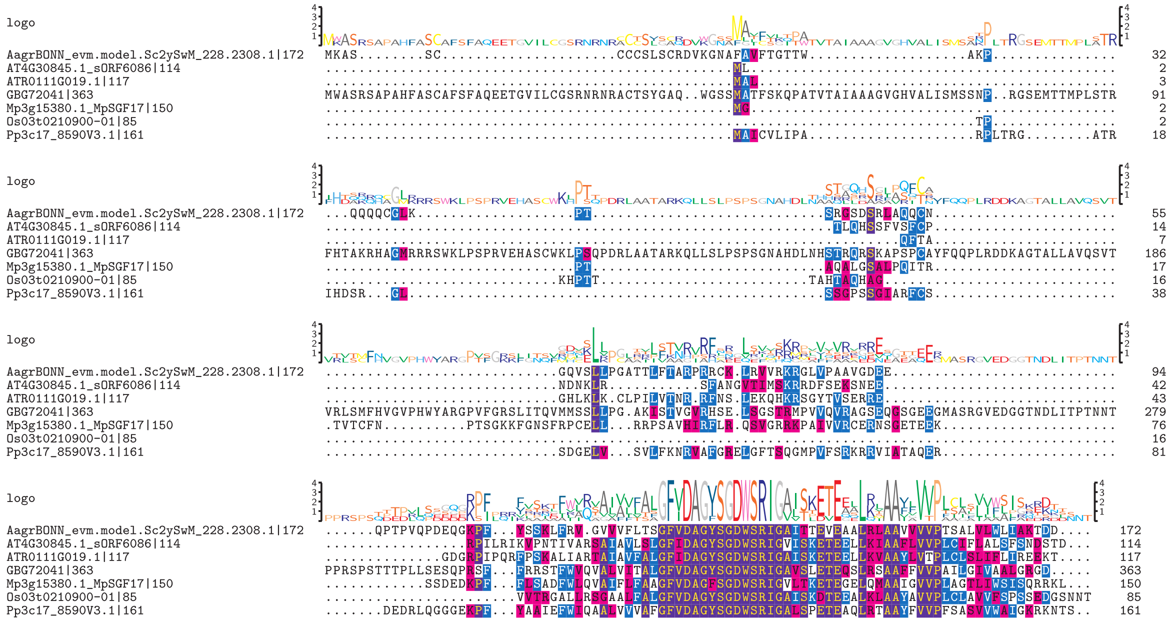

(Continued on next page)

SGF18: ORTHO05D009796

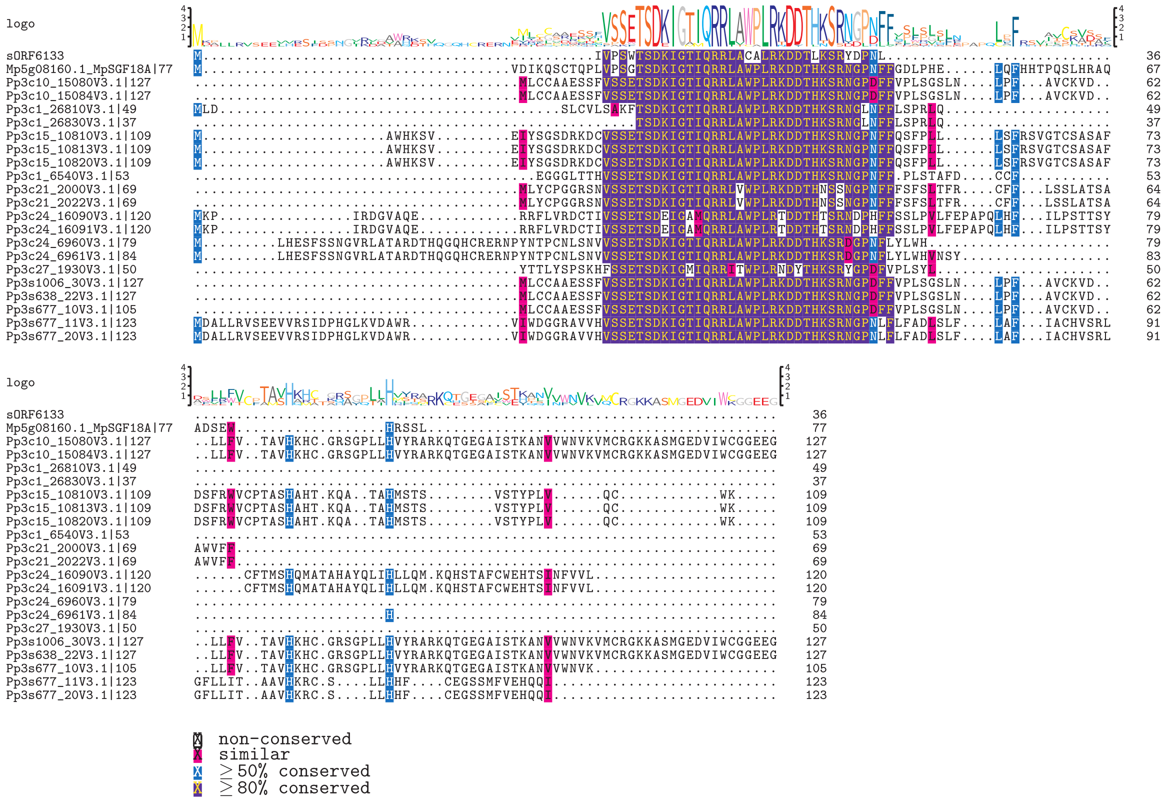

Supplementary Figure S1. Multiple Sequence Alignments of SGFs. Multiple sequence alignments were done by MAFFT (v7.490) with L-INS-i method for SGFs among the following land-plant species: *Arabidopsis thaliana*, *Oryza sativa*, *Sequoiadendron giganteum*, *Amborella trichopoda*, *Selaginella moellendorffii*, *Anthoceros agrestis*, *Physcomitrium patens*, *Marchantia polymorpha*, *Chara braunii*, and *Chlamydomonas reinhardtii*. Lengths of sequences are shown following pipes ("|").

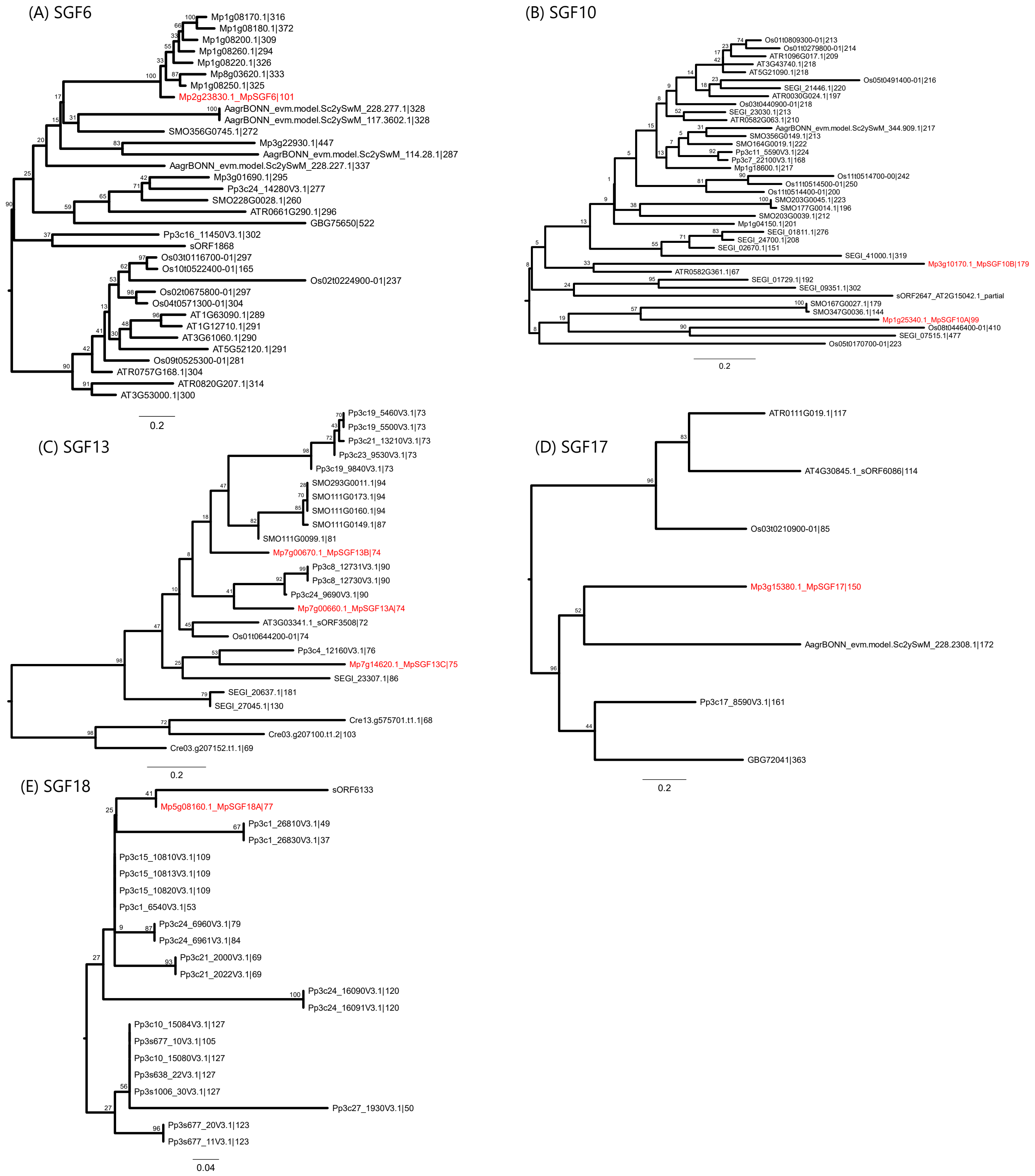

Supplementary Figure S2. Molecular phylogenetic trees of SGFs among plant species: (A) SGF6, (B) SGF10, (C) SGF13, (D) SGF17, and (E) SGF18. Phylogenetic trees were generated using RAxML-NG (v0.9.0), with the substitution models of JTT+G4m, LG+I+G4m, LG+G4m, LG+I+G4m, and JTT+I were used for SGF6, SGF10, SGF13, SGF17, and SGF18, respectively. The trees were visualized using FigTree. Bootstrap values were shown around the nodes. MpSGFs are written in red.

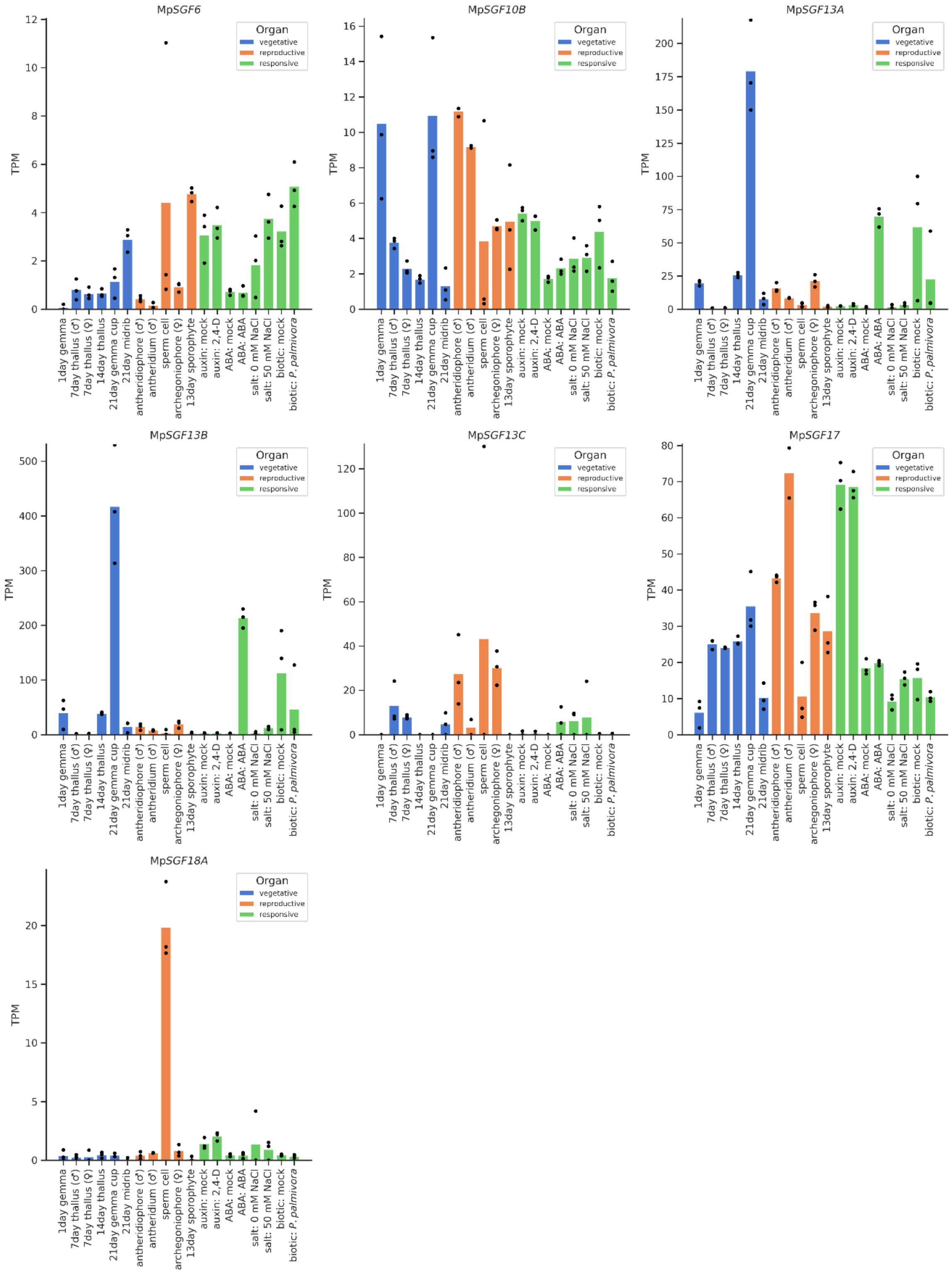

Supplementary Figure S3. Relative expression levels of MpSGFs in various organs of *M. polymorpha*. Data were obtained from Kawamura et al., 2022.

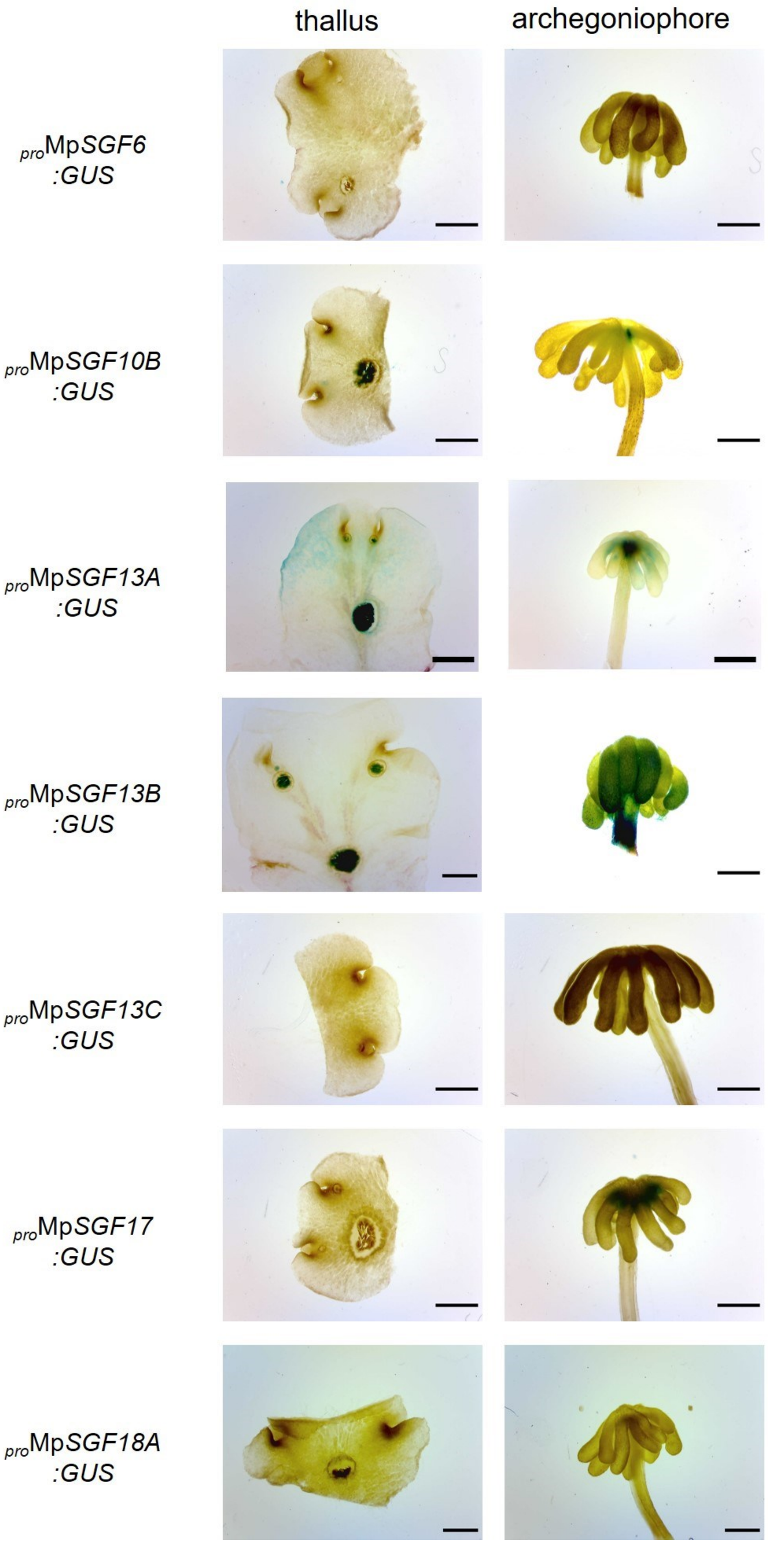

Supplementary Figure S4. GUS reporter assay for MpSGFs in female plants (Tak-2). Three-week-old thalli and archegoniophores were observed for the visualization of promoter activities. Bars = 2 mm.

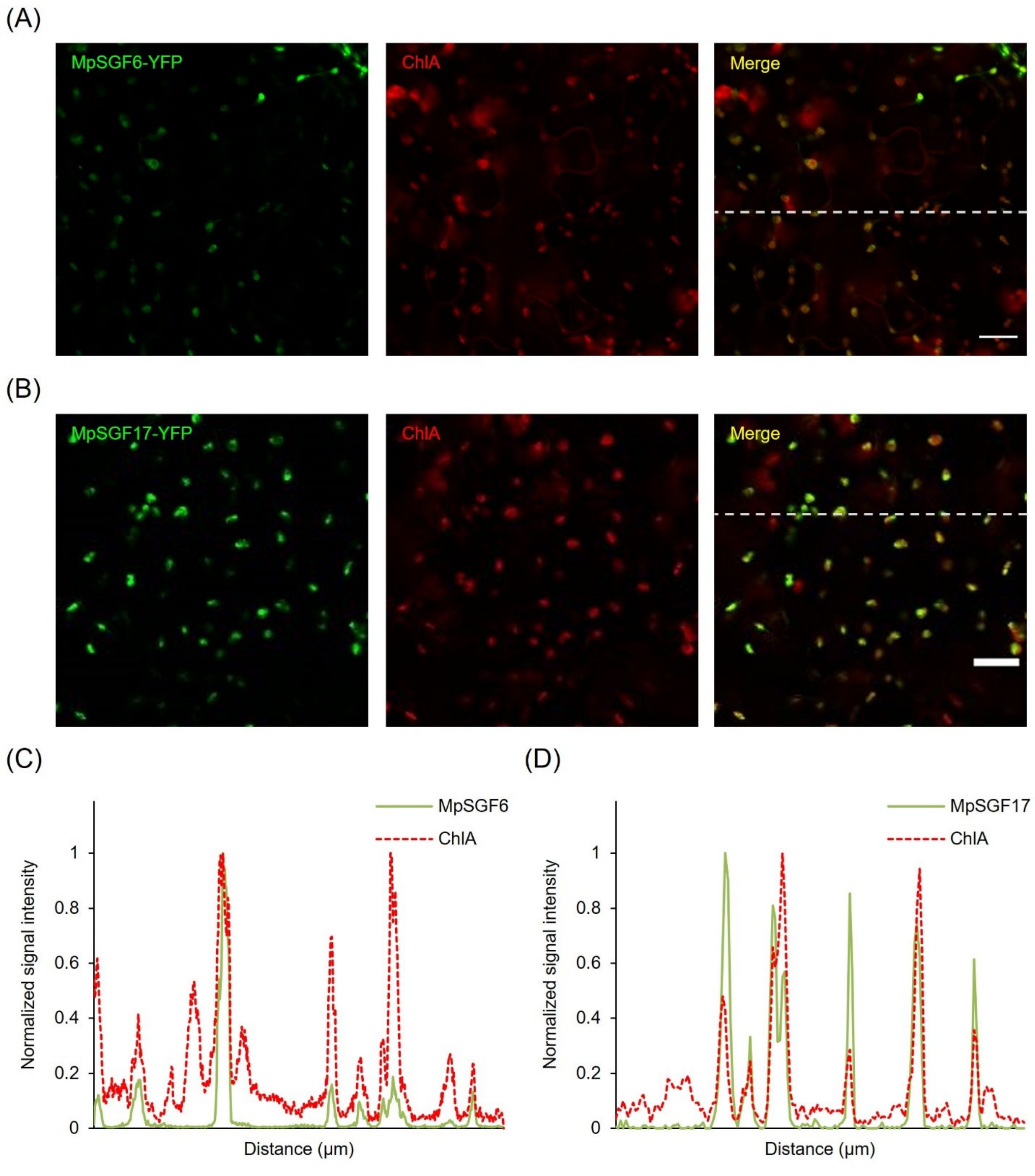

Supplementary Figure S5. Co-localization of MpSGF6 and MpSGF17 with chloroplasts. **(A and B)** Co-localization images of MpSGF6-YFP **(A)** or MpSGF17-YFP **(B)** with chloroplasts in *N. benthamiana*. YFP and chloroplasts were shown in green and red, respectively. Bars = 20 μm. **(C and D)** Min-Max normalized fluorescence intensity of MpSGF6-YFP **(C)** or MpSGF17-YFP **(D)** and chloroplasts. Signal intensities were measured along dashed lines.

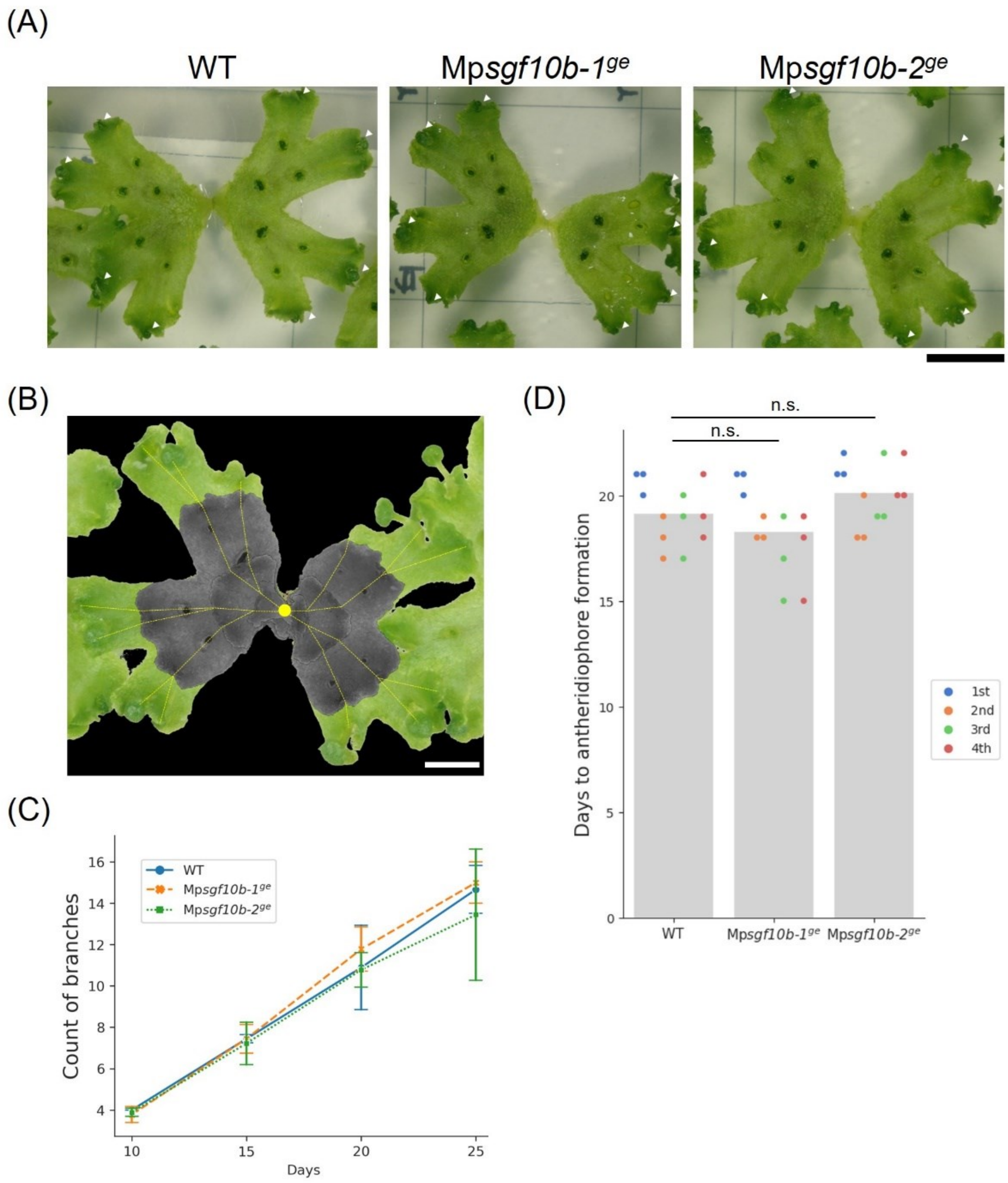

Supplementary Figure S6. Growth phenotypes of Mp*sgf10b* mutants under reproductive-inducing conditions. **(A)** Gross morphology of 25-day-old plants. White arrowheads indicate antheridiophores. Bar = 1 cm. **(B)** Branching trajectory of WT plants during the reproductive inducing condition. Ten-, fifteen-, and twenty-day-old thalli (greyscale) are overlayed on the 25-day-old plant. Apical notches are connected from the initial position of the gemma (yellow dot) by dashed yellow lines. Bar = 5 mm. **(C)** Counts of branches of thalli at the time courses of 10, 15, 20, and 25 days. The branches were counted from the trajectory of apical notches and antheridiophores. $n = 3$ for all lines from four independent experiments. The error bars show the total means ± SD from the grouped averages. (D) Number of days required from the start of incubation to the emergence of gametangiophores. Plants were grown from gemmae under the reproductive-inducing condition. See Materials and Methods 4.1 for detail. n.s. is "no significant" from two-tailed Dunnett's test against WT at a significance level of $P < 0.05$.

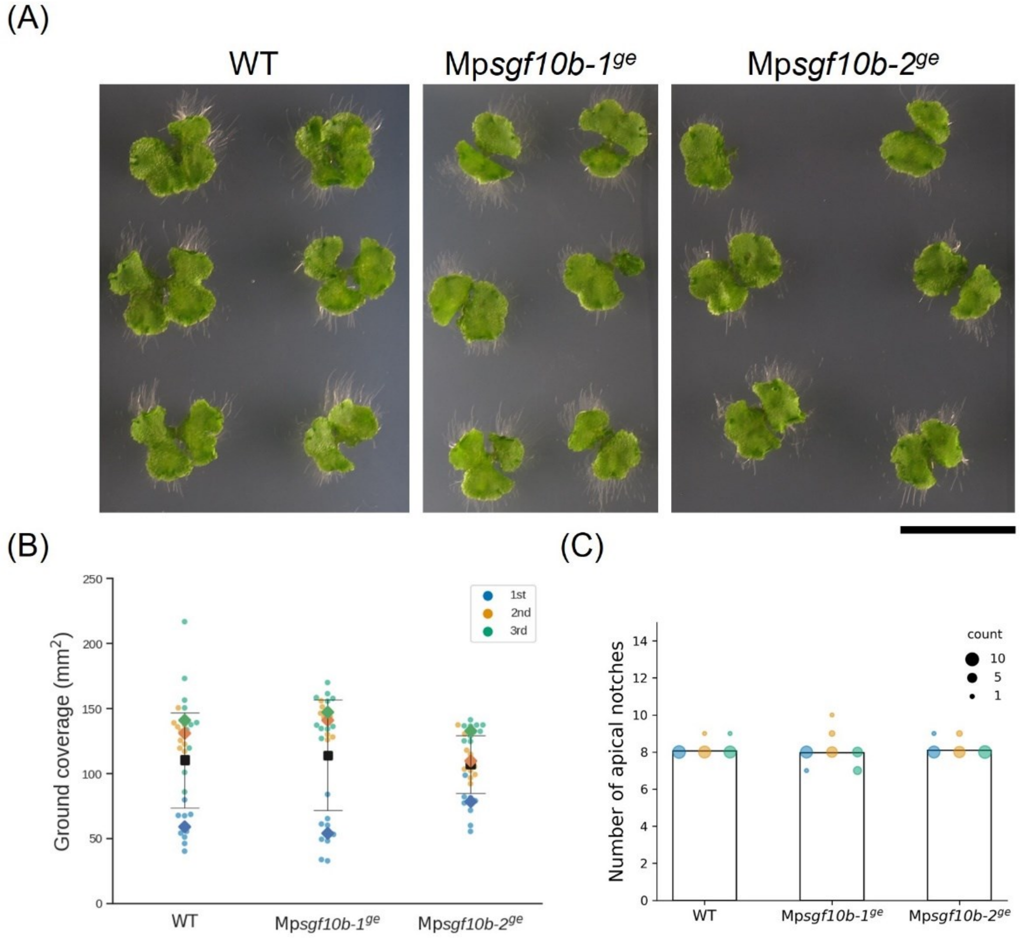

Supplementary Figure S7. Growth phenotypes of Mp*sgf10b* mutants in the vegetative phase. **(A)** Two-week-old plants of WT and transgenic lines of Mp$sgf10b^{ge}$. Bar = 2 mm. **(B)** Ground coverage of 2-week-old-thalli. Areas of thalli were measured with ImageJ. $n = 10$ for all lines from three independent experiments (indicated in distinct colors). Diamonds indicate the averages grouped by each experiment. Black squares and lines show the total means ± SD of the grouped averages. **(C)** The number of apical notches per 2-week-old plant. $n = 10$ for all lines from three independent experiments (indicated in distinct colors). Plants were grown from gemmae under continuous white light at 22°C.

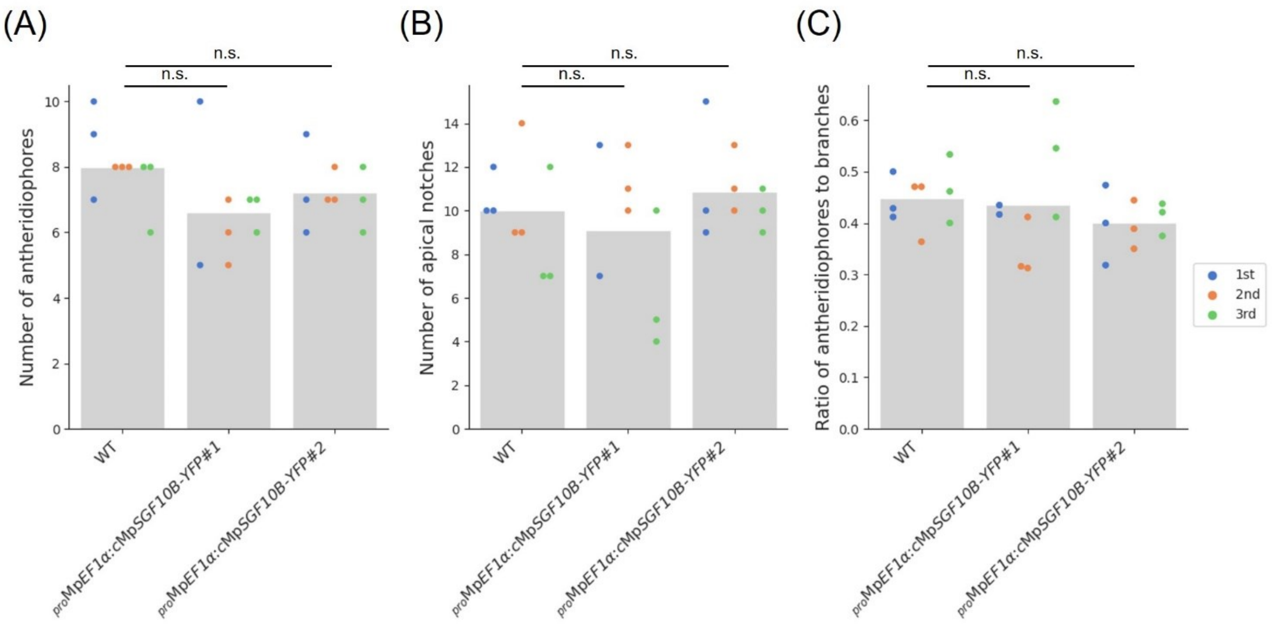

Supplementary Figure S8. Reproductive induction of MpSGF10B-YFP fusion lines. The number of **(A)** antheridiophores and **(B)** apical notches per plant. Thirty-day-old plants grown under the reproductive-inducing condition were observed. Apical notches, in which gametangiophores were not formed, were counted. (C) The ratio of the antheridiophores to the apical notches. n.s. is "no significant" from two-tailed Dunnett's test against WT at a significance level of $P < 0.05$.

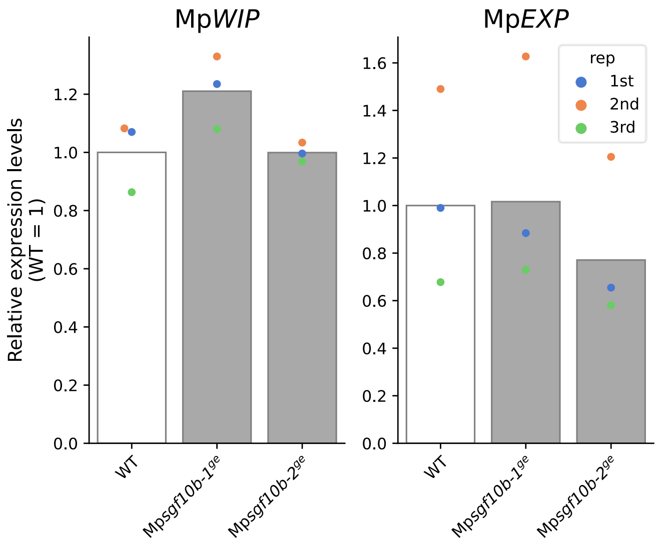

Supplementary Figure S9. Relative expression levels of auxin-responsive genes. Mp*WIP* and Mp*EXP* were used as an auxin-responsive genes (Kato et al., 2017). Five thalli, grown under continuous white light at 22°C for 10 days, were sampled to extract mRNA and synthesize cDNA (for details, see Materials and Methods). Quantitative PCR was performed using TB Green Premix Ex Taq II reagent (Takara Bio) and Light Cycler 96 (Roche).

2 Supplementary Tables

Supplementary Table S1. A tabular of MpSGFs containing results of BLAST, annotations registered in the database (MarpolBase), existence of signal peptide sequence, and prediction of subcellular localization.

Supplementary Table S2. A list from GO enrichment analysis of co-expressed genes with each MpSGFs.

Supplementary Table S3. A table of orthologous genes of SGFs.

Supplementary Table S4. A list of primers and transgenic plants used in this study.
